# Lung Regeneration by Transplantation of Allogeneic Lung Progenitors Using a Safer Conditioning Regimen and Clinical-grade Reagents

**DOI:** 10.1093/stcltm/szab016

**Published:** 2022-02-28

**Authors:** Irit Milman Krentsis, Ran Orgad, Yangxi Zheng, Esther Bachar Lustig, Chava Rosen, Elias Shezen, Sandeep Yadav, Bar Nathansohn Levi, Miri Assayag, Neville Berkman, Harry Karmouty Quintana, Einav Shoshan, Christa Blagdon, Yair Reisner

**Affiliations:** Department of Stem Cell Transplantation and Cell Therapy, MD Anderson Cancer Center, Houston, TX, USA; Department of Immunology, Weizmann Institute of Science, Rehovot, Israel; Department of Immunology, Weizmann Institute of Science, Rehovot, Israel; Department of Stem Cell Transplantation and Cell Therapy, MD Anderson Cancer Center, Houston, TX, USA; Department of Stem Cell Transplantation and Cell Therapy, MD Anderson Cancer Center, Houston, TX, USA; Department of Immunology, Weizmann Institute of Science, Rehovot, Israel; Department of Stem Cell Transplantation and Cell Therapy, MD Anderson Cancer Center, Houston, TX, USA; Department of Immunology, Weizmann Institute of Science, Rehovot, Israel; Department of Neonatology, Edmond and Lily Safra Children’s Hospital, Sheba Medical Center, Tal-Hashomer, Israel; Department of Stem Cell Transplantation and Cell Therapy, MD Anderson Cancer Center, Houston, TX, USA; Department of Immunology, Weizmann Institute of Science, Rehovot, Israel; Department of Stem Cell Transplantation and Cell Therapy, MD Anderson Cancer Center, Houston, TX, USA; Department of Immunology, Weizmann Institute of Science, Rehovot, Israel; Pulmonary Medicine, Hadassah-Hebrew University Medical Center, Jerusalem, Israel; Pulmonary Medicine, Hadassah-Hebrew University Medical Center, Jerusalem, Israel; University of Texas Health Science Center at Houston, Department of Biochemistry and Molecular Biology & Divisions of Critical Care, Pulmonary and Sleep Medicine, Houston, TX, USA; Department of Stem Cell Transplantation and Cell Therapy, MD Anderson Cancer Center, Houston, TX, USA; Department of Stem Cell Transplantation and Cell Therapy, MD Anderson Cancer Center, Houston, TX, USA; Department of Stem Cell Transplantation and Cell Therapy, MD Anderson Cancer Center, Houston, TX, USA; Department of Immunology, Weizmann Institute of Science, Rehovot, Israel; CPRIT Scholar in Cancer Research, Houston, TX, USA

**Keywords:** Lung progenitors, transplantation, allogeneic, rejection, immune suppression, immune tolerance, conditioning

## Abstract

Over the last decades, several studies demonstrated the possibility of lung regeneration through transplantation of various lung progenitor populations. Recently, we showed in mice that fetal or adult lung progenitors could potentially provide donor cells for transplantation, provided that the lung stem cell niche in the recipient is vacated of endogenous lung progenitors by adequate conditioning. Accordingly, marked lung regeneration could be attained following i.v. infusion of a single cell suspension of lung cells into recipient mice conditioned with naphthalene (NA) and 6Gy total body irradiation (TBI). As clinical translation of this approach requires the use of allogenic donors, we more recently developed a novel transplantation modality based on co-infusion of hematopoietic and lung progenitors from the same donor. Thus, by virtue of hematopoietic chimerism, which leads to immune tolerance toward donor antigens, the lung progenitors can be successfully engrafted without any need for post-transplant immune suppression. In the present study, we demonstrate that it is possible to replace NA in the conditioning regimen with Cyclophosphamide (CY), approved for the treatment of many diseases and that a lower dose of 2 GY TBI can successfully enable engraftment of donor-derived hematopoietic and lung progenitors when CY is administered in 2 doses after the stem cell infusion. Taken together, our results suggest a feasible and relatively safe protocol that could potentially be translated to clinical transplantation of lung progenitors across major MHC barriers in patients with terminal lung diseases.

## Graphical Abstract

**Graphical Abstract:**
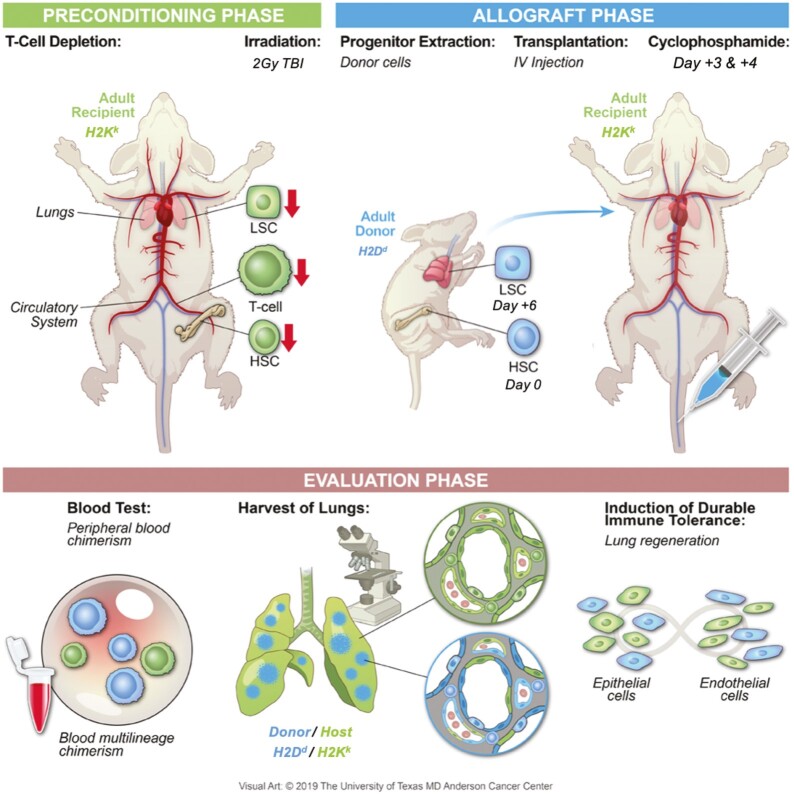

The present study defines the minimal conditioning required for transplantation of lung stem together with hematopoietic stem cells across major MHC barrier, without any post transplant immune suppression. Furthermore it shows that cyclophosphamide, used routinely in clinical transplantation, can be used instead of naphthalene previously employed by the authors to make space for engraftment.

**Significance Statement**

Using clinically approved reagents, the present study demonstrates a relatively safe conditioning protocol enabling lung regeneration by fully mis-matched allogeneic lung progenitors. Furthermore, by combining transplantation of hematopoietic and lung progenitors, this protocol leads to marked hematopoietic chimerism and immune tolerance toward donor antigens, thereby enabling graft maintenance without chronic immune suppression. This proof of concept study paves the way for potential lung regeneration in patients with various lung diseases.

## Introduction

Respiratory diseases are among the leading causes of death worldwide, with more than 5.5 million deaths annually (World Health Organization data for 2020).^1^ Today, the only definitive treatment for these conditions is the replacement of the damaged organ with a lung transplant. However, due to a shortage of suitable organs, many patients die on the transplant waiting list. Therefore, the treatment of lung diseases could greatly benefit from stem cell therapy.

We recently demonstrated in mice that substantial lung regeneration can be attained by I.V. infusion of a single cell suspension of fetal^2^ or adult lungs,^3^ provided that lung stem cell niches in the recipients are vacated by appropriate pre-conditioning, to reduce the competition between the donor and endogenous lung progenitors for the lung niches, in a procedure resembling bone marrow transplantation. To that end, the recipients are initially treated with naphthalene (NA), which induces lung injury and thereby triggers exit from quiescence in lung progenitors of all lineages.^2^ These dividing putative progenitors can then be targeted after 48 hours by exposure to 6Gy total body irradiation (TBI). Thus, transplantation 1 day after irradiation, of syngeneic lung cells from GFP+ or TdTomato+ mouse donors, led to robust regenerative lung “patches” in the recipient’s lungs at 8 weeks post-transplantation, exhibiting full incorporation of donor-derived cells within epithelial and endothelial compartments. Furthermore, the remarkable multi-lineage and durable chimerism levels, attained following the simple infusion of a syngeneic single-cell suspension, was associated with pronounced improvement in lung functions.^2,3^

More recently, we demonstrated that by combining transplantation of hematopoietic and lung progenitors from the same donor, it is possible to attain clear donor-derived patches in the lungs of fully mismatched recipients.^4^ The addition of hematopoietic progenitors in these transplants served to induce durable hematopoietic chimerism and immune tolerance toward donor major histocompatibility complex (MHC), avoiding any need for post-transplant immune suppression in line with several previous studies using hematopoietic stem cells for immune tolerance induction.^5-11^

To further optimize this approach for potential translation to clinical application, we attempted in the present study to replace the use of NA in the first step of lung injury, with clinical-grade reagents such as Busulfan (Bus) or Cyclophosphamide (CY). Furthermore, to minimize the toxicity of the entire protocol, we defined the minimal dose of TBI required for attaining durable hematopoietic as well as lung chimerism in the absence of post-transplant immune suppression.

Taken together, our results provide a very mild, low toxicity protocol for the transplantation of lung progenitors across major genetic barriers, which could be translated to a clinical approach for lung regeneration in different types of lung diseases.

## Materials and Methods

### Mice

Animals were maintained under conditions approved by the Institutional Animal Care and Use Committee at the Weizmann Institute and by the Animal Use Committee at MD Anderson. All the procedures were monitored by the Veterinary Resources Unit and approved by the Institutional Animal Care and Use Committee (IACUC). Mouse strains used included the following: C57BL/6, C57BL/6-Tg (CAG-EGFP)1Osb/J, C57BL/6-TdTomato (B6.129(Cg)-*Gt(ROSA)26Sor*^*tm4(ACTB-tdTomato,-EGFP)Luo*^/J), Balb/c, and C3H/HeJ. All mice were used at 6-18 weeks of age. Mice were kept in small cages (up to 5 animals in each cage) and fed sterile food and acid water. Animals of the same age, sex, and genetic background were randomly assigned to treatment groups. Pre-established exclusion criteria were based on IACUC guidelines and included systemic disease, toxicity, respiratory distress, refusal to eat and drink, and substantial (>15%) weight loss.

For all the experiments, female mice were used as hosts, with a minimum of 5 mice per group.

### Preconditioning of C57BL/6 Host Mice for Allogeneic Transplantation

On day −6 before transplantation C57BL/6 or C3H/Hej female mice were injected with 300 µg/mouse anti-CD4 (BioXCell clone GK1.5) and anti-CD8 antibodies (BioXCell clone YTS169.4). Following T-cell depletion, the recipient mice were treated on day −3 before transplantation with i.p. injection of 200 mg/kg Naphthalene (>99% pure; Sigma-Aldrich), dissolved in corn oil, as previously described^2,3^ or 200 mg/Kg Cyclophosphamide (Sandoz or Baxter) dissolved in PBS, or 25 mg/Kg Busulfan (Otsuka) dissolved in PBS. On day −1 before transplantation, the mice were treated with TBI in an Xrad-320 biological X-ray irradiator. On day 0, the mice were transplanted with different cell populations as described above. On days +3 and +4 post-transplant, mice were treated with Cyclophosphamide (Baxter) (100 mg/kg diluted in PBS, according to the manufacturer’s instructions), injected i.p. Recipient mice were treated with oral Ciprofloxacin delivered through drinking water for 2 weeks post-transplant.

### Harvesting of Mouse BM and Preparation of Single-cell Lung Suspension

Bone marrow cells were prepared for transplantation by crushing long bones of donor mice, using a mixer homogenizer (OMNI). Single-cell suspensions were obtained from enzymatically treated adult and fetal mouse lungs, as previously described.^3^ Briefly, lung digestion was performed by finely mincing tissues with a razor blade in the presence of 1 mg/mL collagenase, 2.4 U/mL dispase and 1 mg/mL DNAseI (Roche Diagnostics) diluted in Ca^+^Mg^+^ phosphate-buffered saline (PBS).

Cells were dissociated by GentalMax (Miltenyi Biotec) incubation for 30 minutes at 37°C. Nonspecific debris were removed by sequential filtration through 100 and 70 µm filters. The cells were then washed with PBS (Ca^+2^ and Mg^+2^ free) with 2% fetal calf serum, antibiotics, and 2 mM ACDC-A. Both lung cells and BM were depleted of CD4 and CD8 cells, using a Miltenyi magnetic microbead separation protocol, according to the instructions of the vendor. Before I.V. injection, the donor cells were filtered again through a 40 µm filter.

Host mice, preconditioned with T cell debulking (TCD), Naphthalene (NA) or CY or Bus and TBI, were transplanted with 8 × 10^6^ C3H/HeJ or BALB adult lung cells, combined with 25 × 10^6^ T-cell-depleted adult donor BM cells. Intravenous cell injection (I.V.) into the tail vein was used for transplantation experiments.

### Removal of T Cells from Lung and BM Cells by MACS

Adult lung single-cell suspension and BM cells were depleted of CD4 and CD8 T cells by Cell Separation (LD) Columns (MACS) (Miltenyi Biotec) in MACS buffer (0.5% bovine serum albumin and 2 mM ACDC-A in PBS), according to the manufacturer’s instructions.

### Flow Cytometry

Cell samples were stained with conjugated antibodies or matching isotype controls according to the antibody manufacturer’s instructions. Antibodies were purchased from e-Bioscience and Biolegend. The complete list of antibodies used in the study is provided in Supplementary Table 1. Data were acquired on BD FACSCanto II or BD LSRFortessa flow cytometer and analyzed using FlowJo software (version 9, or version 10). Chimerism analysis in peripheral blood (PB) was performed 40-60 days post-transplantation.

### Immunostaining

Mice were sacrificed at different time points following transplantation; the lungs were inflated to full capacity with a 4% paraformaldehyde solution introduced through the trachea under constant pressure of 20 cm H_2_O. Then, the lungs were immersed in fixative overnight at 4°C and cryopreserved in 30% sucrose for another 24 hours before snap freezing in isopentane pre-cooled by liquid nitrogen using optimal cutting temperature (OCT) compound (Sakura Finetek USA, Inc. Tissue-Tek.; product code #4583). The samples for frozen sections were harvested and inflated to full capacity with a 1:1 mixture of OCT and PBS and snap-frozen in isopentane pre-cooled by liquid nitrogen. Frozen samples were cut to serial 6-12 µm sections and stained. The list of antibodies used in this study is provided in Supplementary Table S1. All secondary antibodies were purchased from Jackson Laboratories or Abcam. Evaluation of stained samples was performed by upright Olympus BX51 fluorescent microscope with ×10, ×40 air, and ×100 oil objectives, and Olympus digital camera (DP70). Confocal microscopy was performed on an Olympus 3000FV laser scanning confocal microscope, using cell sense software (Olympus). The images were processed, rendered, and reconstructed in 3D in Imaris software (Bitplane AG, Switzerland, http://www.bitplane.com).

### Calculation of Donor-derived Engrafted Area and Donor-derived Foci

Frozen, 12-µm-thick serial sections of chimeric lungs were cut in Leica cryostat at 8 weeks after transplantation, and stained with PE anti-H2K^k^ for C3H donor-derived cells, or APC anti- H2D^d^/ H2K^d^ antibody for Balb/c donor-derived cells. Anti-TdTomato or anti-GFP antibodies were used in the syngeneic transplantation experiments, to detect the donor cells, in combination with other markers, as indicated in the Results section. Serial step sections were taken along the longitudinal axis of the lung. The fixed distance between the sections was calculated to allow systematic sampling of at least 20 sections across the whole lung. Slices were assessed using Olympus fluorescent microscope or Olympus confocal laser scanning microscope, using a ×10 or ×20 objective. Each analyzed image was individually evaluated for validation of the staining pattern before processing. The engrafted area was calculated by Fiji software. The red and green channels were extracted from the RGB image. The percentage of the engrafted area was then calculated from the whole lung tissue. Engrafted areas had high values of red or green intensity, whereas the whole lung tissue had low (autofluorescence) red or green intensity, and the air spaces appeared dark. Whole lung tissue excluding air spaces was detected by applying Gauss blur to the green channel (sigma = 3), setting a low fixed threshold on the blurred image, and further smoothing the edges to remove small artifacts using the dilation and erosion operations. A mask comprising the whole lung tissue was created, with borders delineated in red, and its area measured. The RenyiEntropy global thresholding method was used for calculating the intensity threshold of the high red or green (engrafted) area and to create a mask. The percentage of the high red or green area was calculated from 2 measurements as high red or green area (engrafted)/low red or green area (total tissue) × 100. In most experiments, automated software was used, but in a few instances, manual examination of the slides was required. Nevertheless, in all cases, the reader was blinded to the identity of the sample.^12^

The actual number of donor foci (a group of 6 or more distinct donor cells was defined as a single patch) was calculated by Fiji software (ImageJ, https://fiji.sc). The average size of each donor patch was calculated as described before,^2-4^ using random slices from host mice transplanted with lung cells with BM, and stained with PE anti-H-2K^k^ Ab in the first model, or APC anti-H-2K^d^/H-2D^d^ in the second model, following the various conditioning protocols.

### Assessment of Airway Hyper-responsiveness

Mice were anesthetized using Avertin and ventilated with a FlexiVent apparatus (SCIREQ, Montreal, QC, Canada). Two models of respiratory mechanics were used to assess lung resistance (R): the linear first-order single compartment model and the constant-phase model. All data points were collected with FlexiVent software (SCIREQ). Results were analyzed using PRISM software^3,13-16^

### Statistical Analysis

Differences between groups were evaluated using 1-way variance analysis (ANOVA) and Dunnett’s post-hoc test for calculating the *P* value between the different groups, using Prism software. For each data set, mean ± SD or mean± SEM was calculated and is presented in the Results section of the main text. *P* value ≤ .05 was considered statistically significant.

## Results

### Potential Replacement of NA with Clinical Grade Reagents in the Conditioning Regimen Before Lung Cell Transplantation in Syngeneic Recipients

**Figure 1.**
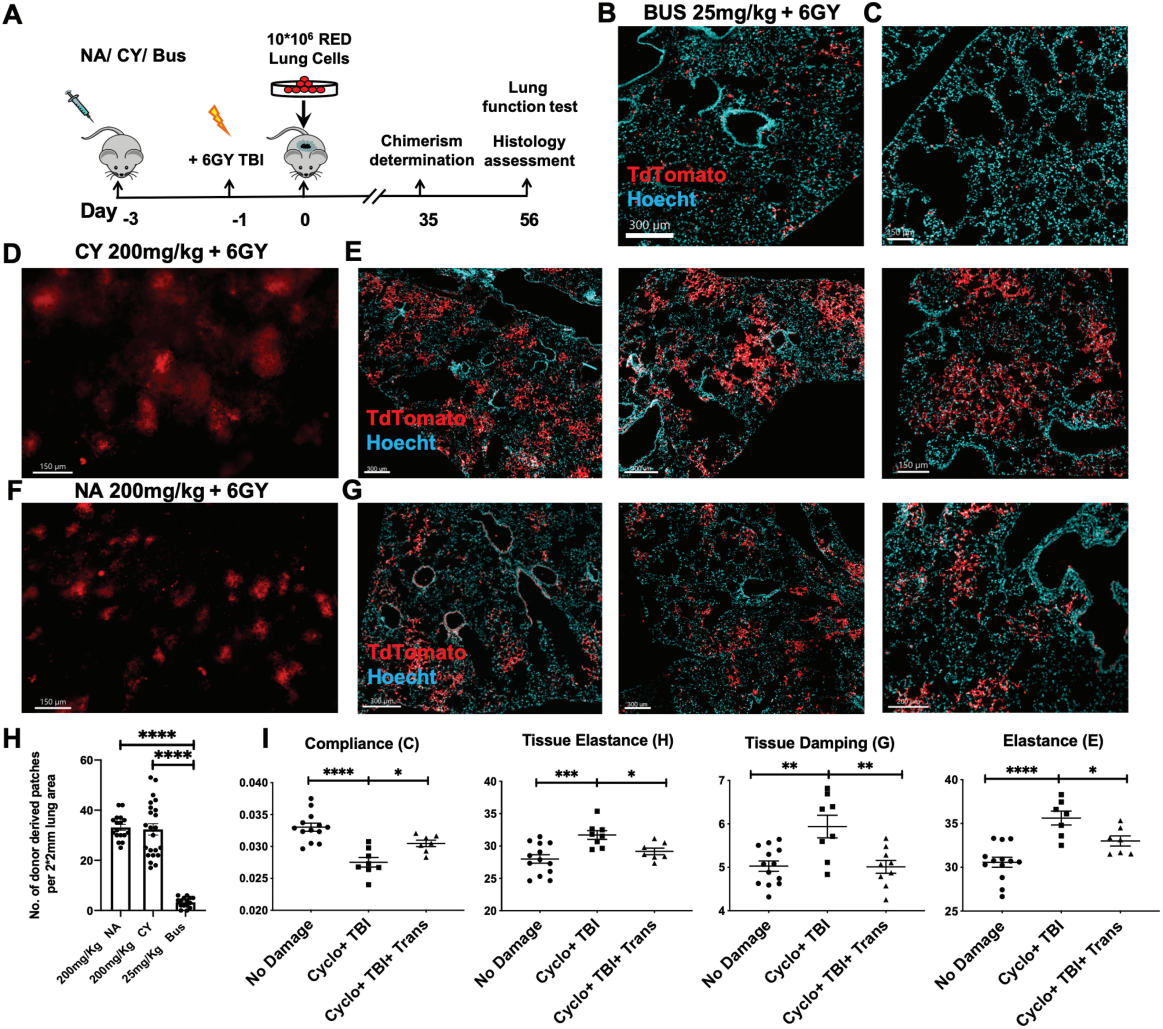
Donor-derived patches in recipients of TdTomato+ lung progenitors following conditioning with different reagents. (**A**) C57BL/6 mice were pre-conditioned with either 200 mg/kg of NA or CY, or 25 mg/kg of Bus, IP, 3 days before transplantation, and irradiated with 6GY TBI 2 days after conditioning. After 1 day, the mice were transplanted with 10 × 10^6^ C57BL-TdTomato (Red) lung single cells. (**B-G**) Immunohistological analysis of donor-derived lung patches 8 weeks post-transplantation after pre-conditioning with a protocol including Busulfan (B,C), CY (D,E), or NA (F,G) (scale bars =150 µm to 300 µm, as indicated). D and F show whole mount host lung tissue. B,C, E, and G show typical IHC staining of lung tissues from different recipient mice. (**H**) Summary of morphometric analysis - donor-derived patches were counted, in a 2 × 2 mm field, as described in Methods; 3-5 fields per mouse and 4-5 mice per group were analyzed. Mean and SEM results are shown. One-way ANOVA test was used for statistical analysis (*P* < .0001 (∗∗∗∗), *N* = 15-23). (**I**) Functional analysis measuring compliance, tissue damping, elastance, and tissue elastance at 8 weeks post-transplantation in mice receiving CY as pre-conditioning treatment compared to untreated mice and mice preconditioned but not receiving a transplant. (Mean ± SEM, *n* = 7-13, *P* = .033(∗), .002(∗∗), .0002 (∗∗∗), .0001 (∗∗∗∗)).

To define the possibility of replacing NA with clinical-grade reagents, we tested whether CY or Bus, routinely used in HSCT, could enable successful engraftment and colonization of donor-derived lung progenitors. As shown schematically in Fig. 1A, C57BL/6 host mice were administered NA (200 mg/kg), CY (200 mg/kg), or Bus (25 mg/Kg) by IP injection 3 days before transplantation. After 48 hours, the mice were treated with sub-lethal 6Gy TBI, and 1 day later were transplanted with 10 × 10^6^ lung single-cell suspension from donors expressing TdTomato (red) fluorescent protein.

Notably, while Bus-based conditioning was found to induce inferior engraftment with only a small number of scattered red cells in the recipient's lungs (Fig. 1B,C), conditioning with CY led to robust levels of donor-derived patches (Fig. 1D,E), similar to those found when using NA in the conditioning protocol (Fig. 1F,G). Quantitative morphometric analysis performed at 8 weeks post-transplant revealed a comparable number of donor-derived patches in the lungs of recipient mice treated with NA or CY (32.35 ± 2.3 and 33.12 ± 1.2 per 2 × 2 mm field, respectively), while conditioning with Bus resulted in only 3.27 ± 0.5 donor-derived patches (Fig. 1H). Likewise, as can be seen in Fig. 1I, functional assays measuring elasticity, tissue damping, and lung compliance revealed the marked level of lung injury repair in mice conditioned with CY before 6Gy TBI. Thus, CY plus 6Gy TBI, similarly to our previous finding using NA plus 6Gy TBI, can be used effectively for clearing the lung stem cell niches to overcome stem cell competition between donor and host lung progenitors.

Furthermore, similarly to our previous results using NA in the conditioning protocol,^2,3^ immuno-histological analysis revealed that the donor-derived patches obtained in mice preconditioned with CY plus 6Gy TBI, exhibited robust expression of both epithelial and endothelial markers (Supplementary Fig. S1).

### Transplantation of Lung Progenitors Across Major Genetic Barrier Following Pre-Conditioning with a CY-based Protocol

**Figure 2.**
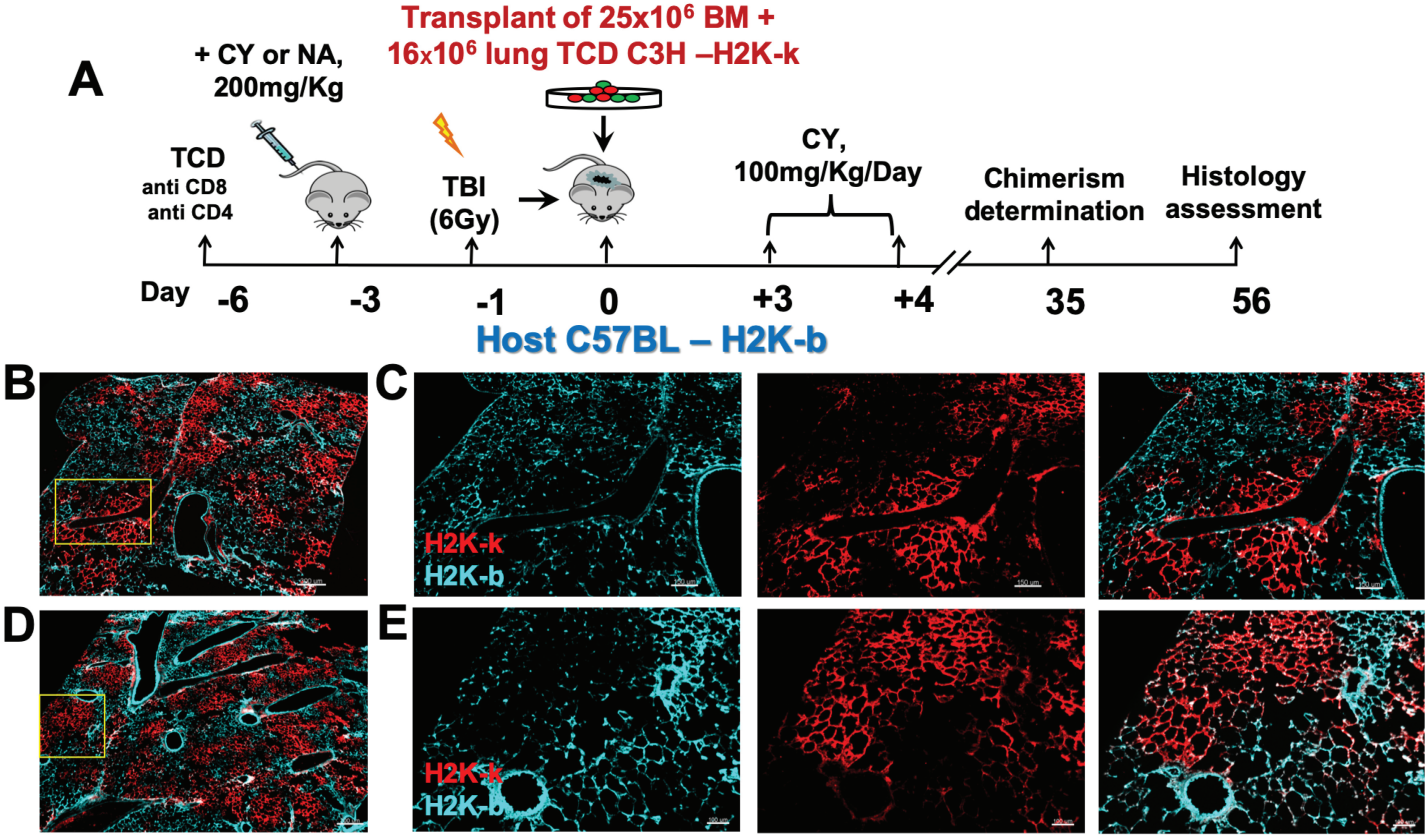
Replacement of NA with CY in the conditioning of allogeneic recipients of lung cell transplantation leads to similar high levels of donor-derived patches. (**A**) Schematic presentation of the transplantation protocol. (**B-E**) Immunostaining of donor-derived patches in recipients of allogeneic lung cells at 8 weeks post-transplantation (H-2Kk, donor, red, and cyan for host type cells ) of 16 × 106 T-cell-depleted adult donor lung cells together with 25 × 106 T-cell-depleted BM cells following NA based (B,C) or CY based (D,E) conditioning (B and D show large field [scale bar 300 µm]. Inset denoted in these fields was analyzed at higher magnification [scale bar 100 µm] depicting single staining for host and donor cells and merge of both in C and E).

We previously demonstrated that transplantation of lung progenitors can be achieved in fully mis-matched recipients by combining donor-derived lung progenitors with BM cells from the same donor, enabling induction of durable immune tolerance toward donor cells without any need for post-transplant immune suppression.^4^ Therefore, based on our present findings in syngeneic recipients that CY can replace NA in the conditioning protocol, we interrogated this possibility in allogenic recipients. As shown schematically in Fig. 2A, our previously described protocol for allogenic lung cell transplantation comprises treatment of C57BL/6 mice with anti-CD4 and anti-CD8 antibodies on day −6, NA treatment on day −3, 6GY TBI on day −1, transplantation of T-cell-depleted BM cells (25 × 10^6^) plus 16 × 10^6^ T-cell-depleted lung cells from C3H donors on day 0, followed by treatment with CY on day+3 and +4 post-transplant. Thus, we next tested the possibility of replacing NA with CY in this allogenic transplantation protocol (Fig. 2A). As shown in Fig. 2B-E, 8 weeks after transplantation, the recipient mice exhibited a similar high level of donor-derived allogeneic patches (stained for H-2K^K^ expression) in the group conditioned with NA (Fig. 2B,C) or with CY (Fig. D,E).

**Figure 3.**
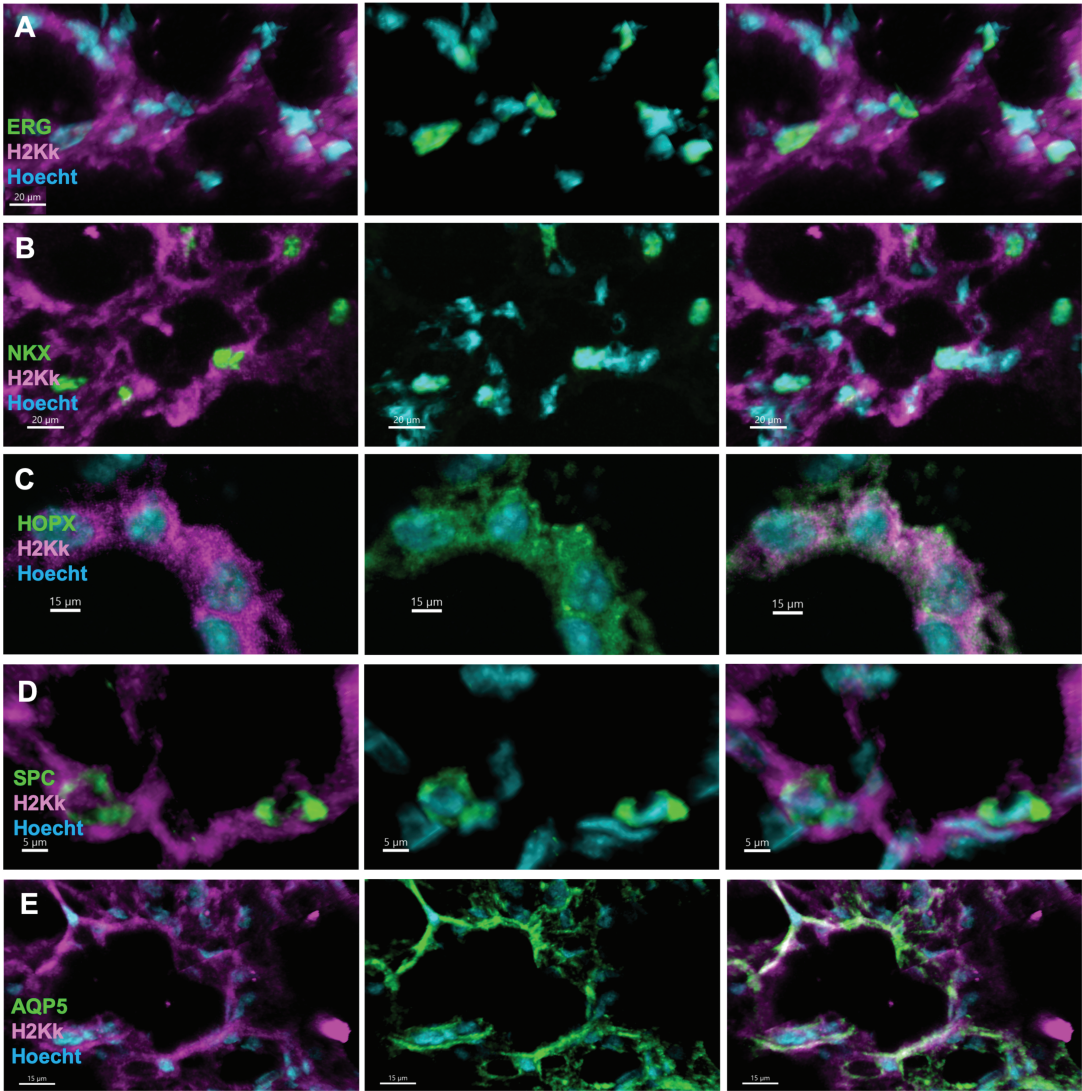
Representative lung immunostaining of donor derived patches 8 weeks following transplantation of allogenic lung cells, depicting integration of donor-derived cells into both the epithelial and endothelial compartments. (A) Left-Double staining for donor derived cells stained for H-2Kk (magenta) and nuclei (Hoecht, cyan). Middle- double staining for the endothelial nuclear marker ERG and for nuclei (cyan). Right- Merge of triple staining for donor derived ERG positive cells. B)Left- double staining for H-2Kk (magenta) and the epithelial cell marker NKX2.1 (green). Middle-double staining for NKX2.1 (green) and nuclei (cyan). Right- Merge of triple staining for donor derived NKX2.1 positive cells. (C) Left- Double staining for donor derived cells stained for H-2Kk (magenta) and nuclei (cyan). Middle- double staining for the epithelial marker HOPX (green) and for nuclei (cyan). Right- Merge of triple staining for donor derived HOPX positive cells. (D) Left-double staining for H-2Kk (magenta) and the AT2 alveolar cell marker SPC (green). Middle-double staining for SPC (green) and nuclei (cyan). Right- Merge of triple staining for donor derived SPC positive cells. (E) Left- Double staining for donor derived cells stained for H-2Kk (magenta) and nuclei (cyan). Middle- double staining for the AT1 alveolar cell marker AQP-5 (green) and for nuclei (cyan). Right- Merge of triple staining for donor derived AQP-5 positive cells. Representative images of *N* = 12 mice, from 4 experiments (Scale bars = 5-20 µm, as marked).

Furthermore, similarly to previous results using the NA-based protocol, CY-based conditioning led to patches exhibiting both donor-derived endothelial cells stained for ERG, CD31, and SOX17 (Fig. 3A and Supplementary Fig. S2), and epithelial cells stained for NKX2.1+ (Fig. 3B) and HOPX+ (Fig. 3C), E-Cadherin+ and wide spectrum Cytokeratin+ (Supplementary Fig. S3). These epithelial cells include alveolar AT2 cells stained for SPC (Fig. 3D), SPB and LAMP3 (Supplementary Fig. S4), and alveolar AQP5+ AT1 cells (Fig. 3E).

### Defining the Minimal Dose of TBI for Allogeneic Lung Cell Transplantation

As our allogenic protocol (Fig. 2A) also includes treatment with CY on days 3 and 4 post-transplant aimed at overcoming rejection, and since CY is a chelating agent like TBI capable of killing dividing cells, we wished to determine whether TBI dose could be reduced in the protocol. To this end, we slightly modified the mouse model based on our previous experience with a mouse model for T-cell-depleted BM allograft rejection.^8^ Thus, in line with this early study, we used Balb/c (H-2^d^) donors and C3H (H-2^k^) mice as recipients.

**Figure 4.**
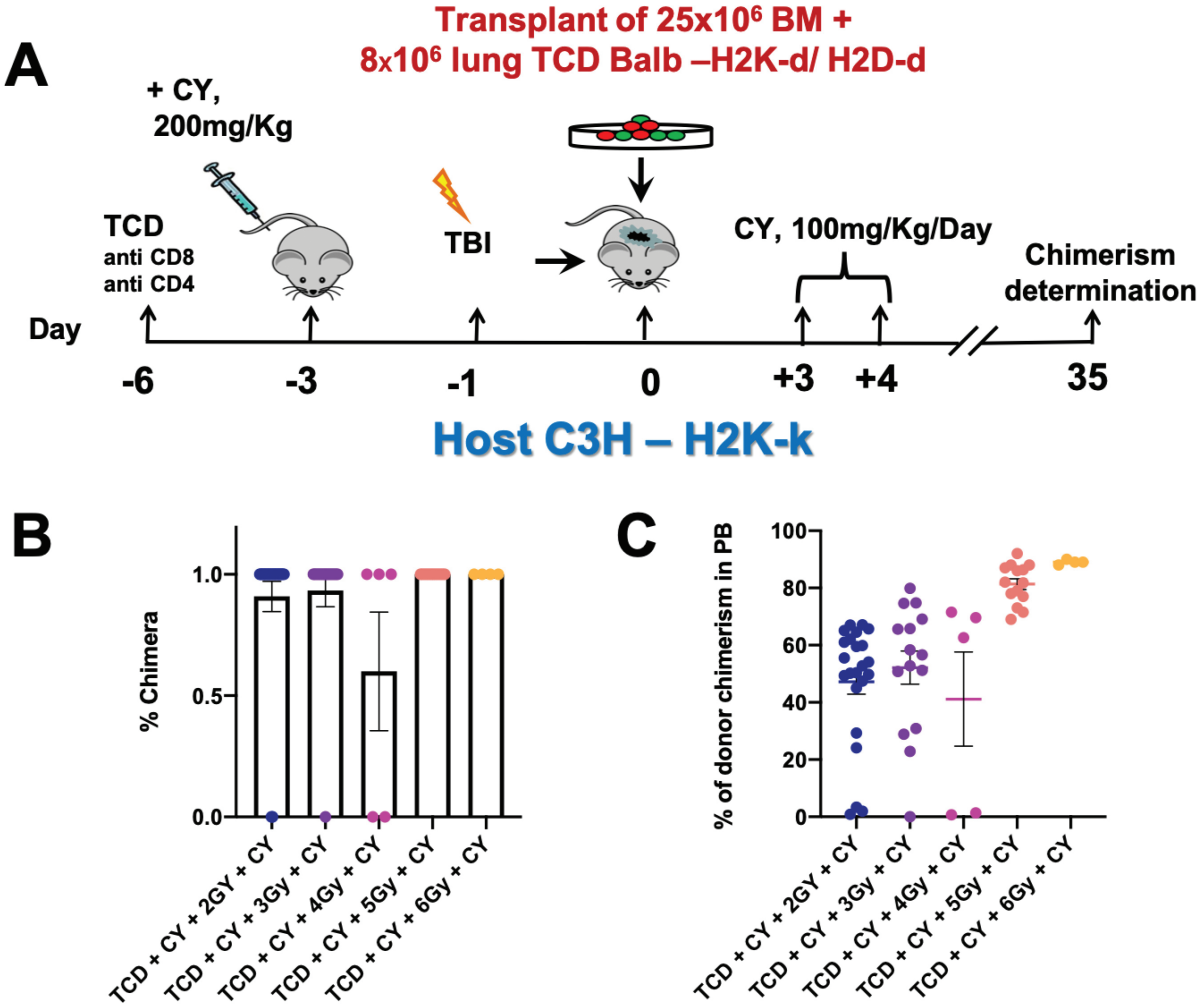
Hematopoietic PB chimerism following reduced doses of TBI in the conditioning protocol before allogenic transplantation of lung plus BM cells from the same donor. C3H/Hej mice were conditioned by T-cell debulking on day −6, CY on day −3, different doses of TBI on day −1, transplantation of 25 × 106 T-cell-depleted BM cells from Balb/c donors together with 8 × 106 lung cells on day 0, and CY on days +3 and +4- as shown schematically in **A**. (**B**) Percent chimeric mice in each of the groups receiving different doses of TBI (*n* = 5-8 mice per group, showing mean and SEM). (**C**) Percentage of donor chimerism in the PB of the different groups. Each dot represents PB chimerism of an individual mouse (*n* = 5-22 mice, *N* = 1-3 pooled experiments).

**Figure 5.**
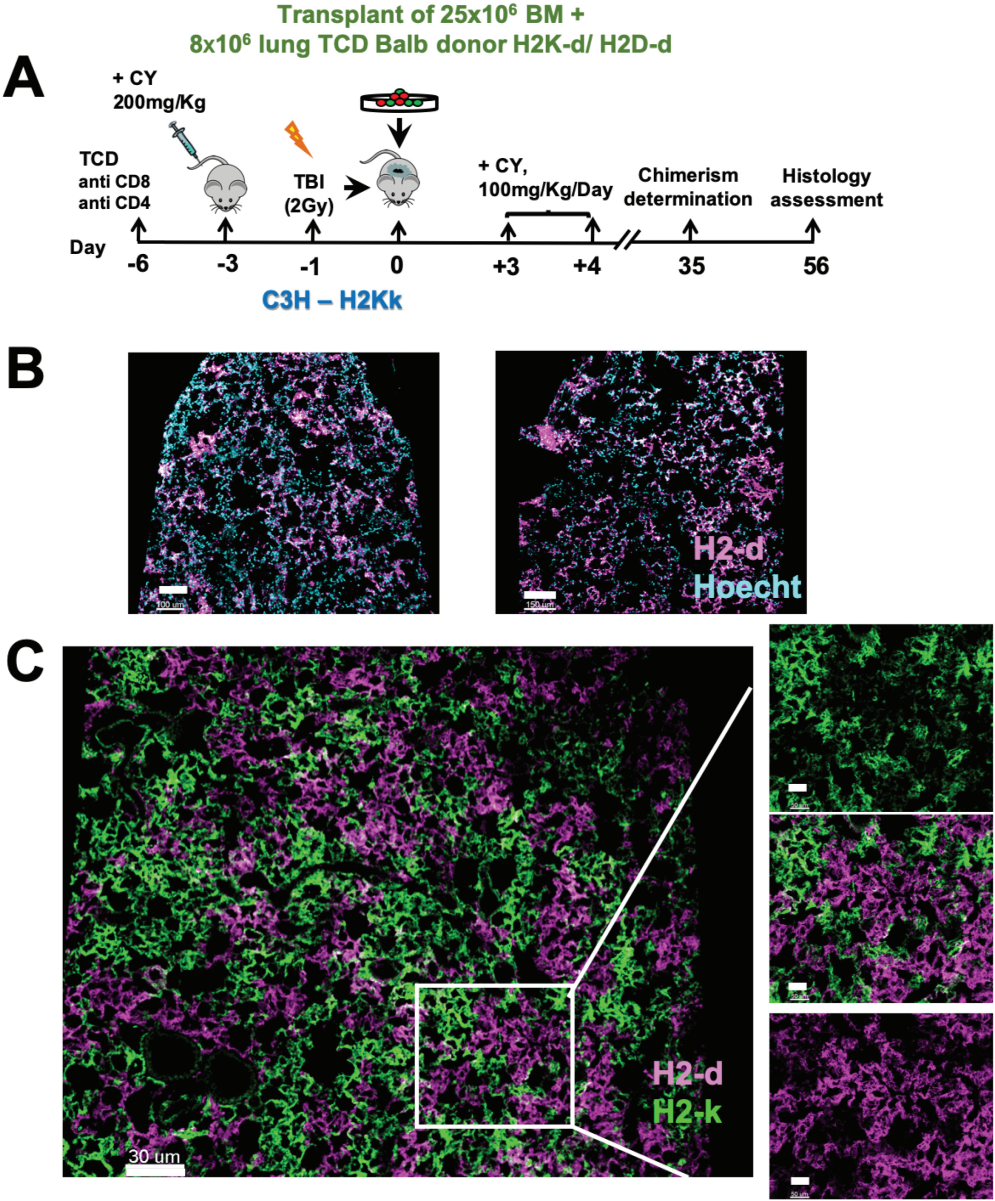
Lung chimerism in mice transplanted following conditioning with a CY-based protocol including 2GY TBI. (**A**) Schematic representation of the transplantation protocol. (**B,C**) Immunohistological analysis of donor-derived patches (magenta) under low (B) (bar size = 100 µm right and 150 µm left), and high (C) magnification, together with the host lung tissue, in green (bar size =30 µm left and 50 µm right).

As shown in Fig. 4A, using different doses of TBI, we initially found that hematopoietic chimerism required for the establishment of durable immune tolerance is retained even when using a low dose of 2GY TBI. Thus, the percent of mice exhibiting hematopoietic chimerism in the peripheral blood (PB) and the average level of PB chimerism in each mouse at 8 weeks post-transplant were 90 ± 6 and 47.23 ± 4.4, respectively, compared to 100 ± 0 and 89 ± 0.4, respectively, in mice conditioned with 6 GY TBI (Fig. 4B,C). Notably, using a protocol comprising this low level of TBI (shown schematically in Fig. 5A), we also found marked lung chimerism at 8 weeks post-transplant, as measured by immunohistological staining of donor-derived patches (Fig. 5B,C).

### Further Reduction of the Conditioning Toxicity by Elimination of CY Treatment Before TBI

**Figure 6.**
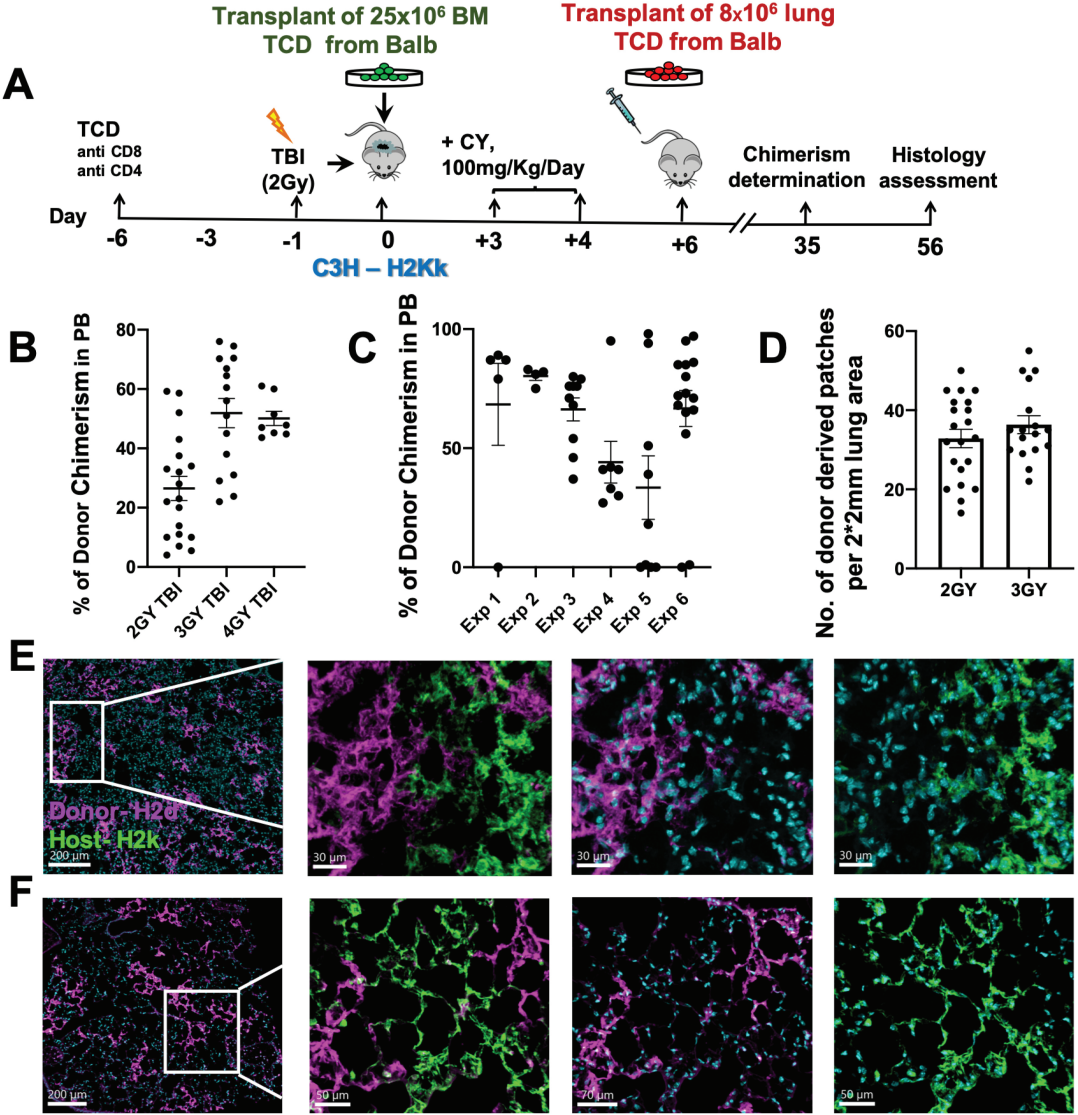
Successful lung cell engraftment following a less intensive conditioning protocol without inclusion of CY before TBI. (**A**) Schematic representation of the modified conditioning protocol. (**B**) Percentage of donor chimerism in the PB following conditioning with different doses of TBI. Each dot represents PB chimerism of an individual mouse. Graphs show mean and SEM. (**C**) Percentage of donor chimerism in the PB of mice conditioned with 2GY TBI in different independent experiments. Each dot represents PB chimerism of an individual mouse, showing mean and SEM (*N* = 6, *n* = 4-15 in each experiment). (**D**) Morphometric analysis of donor-derived patches 8 weeks after transplantation of T-cell-depleted lung cells into allogeneic mice conditioned as described in A with different doses of TBI (*n* = 15-20 mice per group pooled from experiments no. 3, 4 and 6 shown in C. Lung chimerism was tested in mice exhibiting PB chimerism, showing mean and SEM). (**E,F**) Typical donor-derived patches found in mice conditioned with the protocol shown in A and receiving 2GY (E), or 3GY (F) TBI, respectively. Donor (Magenta) and host (green) lung cells are labeled by MHC staining and cyan was used for nuclei (scale bar = 200 µm and 30 µm for the inset images).

As TBI itself can induce an injury response, which might in turn lead to exit of host lung progenitors from quiescence, we hypothesized that in such a case, the use of CY following transplantation of the BM cells might be sufficient to eliminate the responding endogenous lung cells seen after lung injury. Thus, we investigated the possibility of administrating the lung progenitors on day 6 after BMT, namely, 2 days after completion of CY treatment, administered on days 3 and 4 post-BMT (Fig. 6A). Initially, we re-tested different TBI doses including 2, 3, and 4 GY TBI, and as shown in Fig. 6B, marked bone marrow-derived hematopoietic chimerism was found even after conditioning with the low dose of 2GY TBI. Typical examples of the robust lung chimerism achieved following this conditioning protocol with 2-3 GY TBI in 6 independent experiments are shown in Fig. 6C.

Likewise, the quantitative morphometric analysis revealed high levels of donor-derived lung patches even when using 2 GY TBI (Fig. 6D).

**Figure 7.**
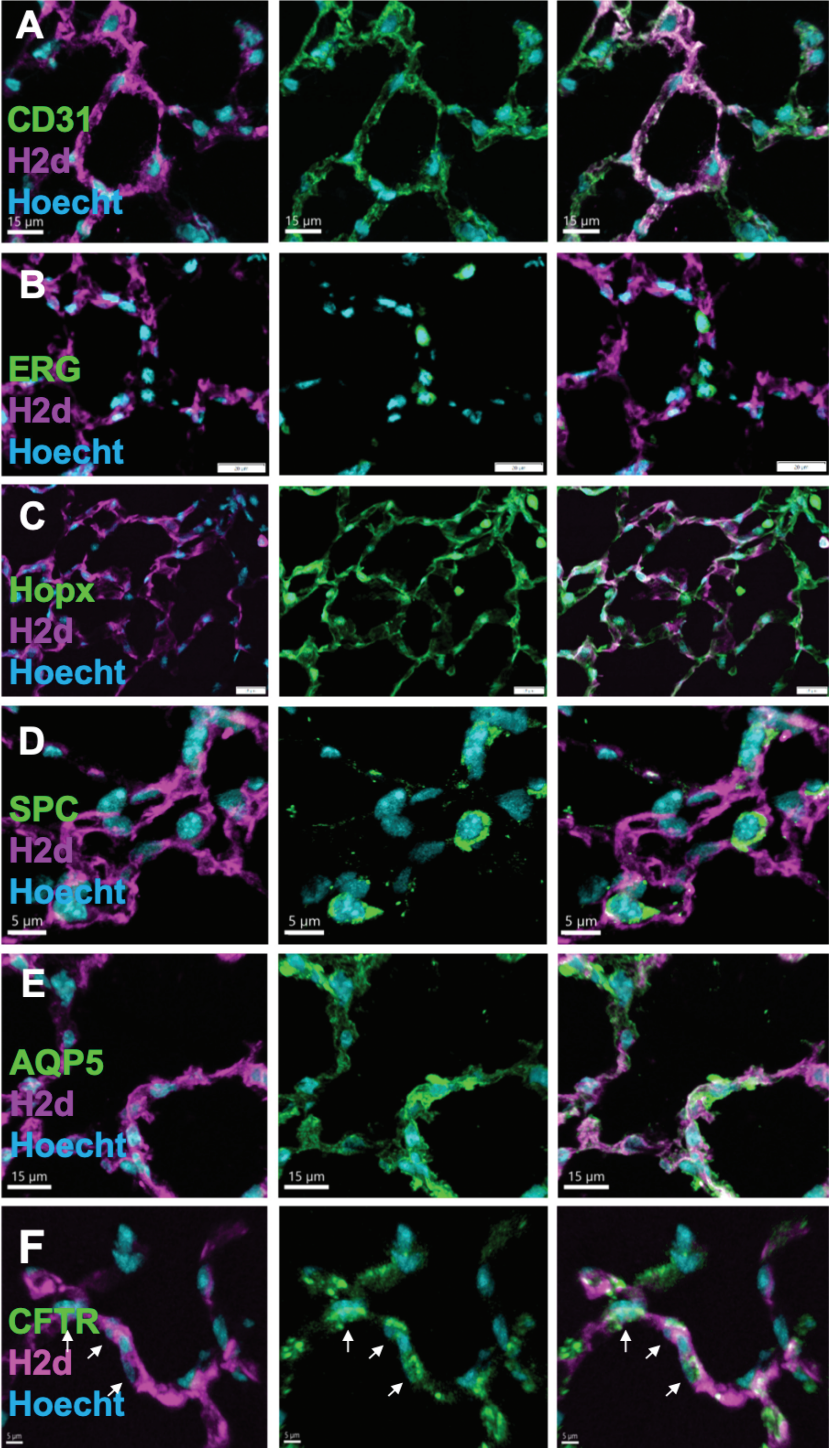
Donor-derived lung patches following a less intensive conditioning protocol, without CY before TBI, contain endothelial and epithelial cells. (**A,B**) Donor-derived (H-2Kk; magenta) lung “patches” comprise cells positive for the endothelial cell surface marker, CD31 (A), and ERG (B) (Green). (**C**) Donor-derived alveolar lung cells positive for the epithelial marker, HOPX (Green). (**D**) SPC staining of donor-derived AT2 alveolar cells (Green). (**E**) AQP-5 staining of AT1 alveolar cells. (**F**) Staining of cystic fibrosis transmembrane conductance regulator (CFTR) (scale bar = 5 µm) (Green). Representative images of *N* = 12 mice, from 4 experiments. Scale size bar = 5-20 µm.

Notably, as found for the more intensive conditioning protocols, these donor-derived lung patches exhibited endothelial cells stained for CD31 (Fig. 7A) and ERG (Fig. 7B), alveolar epithelial cells stained for HOPX (Fig. 7C), SPC (Fig. 7D), and AQP-5 (Fig. 7E), as well as CFTR bronchial cells (Fig. 7F).

## Discussion

We previously described a new approach for the successful transplantation of lung progenitors across major MHC barriers.^4^ This approach is based on 2 basic elements: (1) induction of durable immune tolerance by virtue of megadose hematopoietic stem cell transplantation, and (2) overcoming lung stem cell competition mediated by the endogenous lung progenitors, using adequate conditioning with NA, and subsequent 6GY TBI.

In the present study, we show that it is possible to replace NA in the conditioning protocol with CY, which is commonly used in the treatment of several diseases.^17-22^ Furthermore, our investigation of the potential use of lower doses of TBI enabled us to attain marked lung chimerism even at a low dose of 2GY TBI. Thus, we now found that lung injury with a chelating agent such as CY can effectively induce exit from quiescence as found for NA, and can therefore be used to overcome stem cell competition between donor and host lung progenitors. Furthermore, our ability to achieve marked lung chimerism when reversing the order of CY and TBI administration, both of which cause DNA damage and eliminate dividing cells, suggests that these agents are interchangeable for the induction of exit from quiescence, as well as for ablation of dividing host lung progenitors.

Thus, our results offer a relatively very safe protocol for successful transplantation of lung progenitors, comprising debulking of T cells on day −6, 2Gy TBI on day −1, a transplant of megadose T-cell-depleted BM on day 0, treatment with CY on days +3 and +4, and transplantation of lung cell progenitors on day +6. The tolerance induction arm in this protocol is based on our previous finding that a similar protocol without the infusion of lung progenitors can induce durable immune tolerance in mice, and this proof of concept has recently been translated successfully for the treatment of patients with hematological malignancies.^8^ Thus, our surprising finding that this mild conditioning can sufficiently reduce the stem cell competition between endogenous and donor lung progenitors, offers a much safer and clinically feasible modality for lung regeneration by transplantation of mis-matched lung progenitors.

Our finding of marked alveolar regeneration including AT2 cells after transplantation suggests that this approach could be attractive for the treatment of patients with idiopathic pulmonary fibrosis (IPF). Likewise, our results showing donor-derived CFTR-positive cells after transplantation suggest potential application in CF patients. Further studies in appropriate preclinical models for IPF and CF are warranted. Notably, considering that in IPF patients endogenous lung progenitors might be progressively ablated, it is possible that the toxicity of the conditioning protocol could be further reduced compared to that employed in normal recipients. In contrast, for patients with CF in whom mutated endogenous progenitors need to be replaced by donor-derived progenitors, we envision that the conditioning required might be similar to that used in normal recipients. However, further studies on large animal models of CF are warranted. The application of this approach for lung regeneration in chronic obstructive pulmonary disease (COPD) or in bronchiolitis obliterans syndrome after hematopoietic stem cell transplantation might be more difficult, considering the massive damage of the lung progenitor niches in these diseases, although further studies in appropriate preclinical models are warranted.

Considering that the major source of lung tissue is currently limited to cadaveric donors, and based on our previous finding that lung cell transplantation can be effectively performed with cryopreserved lung single-cell suspensions, it is potentially feasible to harvest lung cells from different cadaveric donors and cryopreserve them in a procedure akin to cord blood banking. Notably, for immune tolerance induction, it will be crucial to also bank T cell-depleted bone marrow cells from each donor, using CD34 selection^23^ or CD3/CD19 depletion with magnetic beads.^24^ For cryopreservation, it is possible to use well-established techniques.^25,26^ However, if due to technical issues, the latter will not be possible for a particular donor, the transplantation procedure will be limited in such a case to transplantation in the context of chronic immune suppression.

Furthermore, preliminary results suggest that it might be possible in the future to expand in-vitro the patch forming lung progenitors, so that a sufficient number of cells may be obtained from biopsies of live donors. Clearly, when using family members as donors, hematopoietic stem cells will be easily available for the induction of tolerance.

In conclusion, our results suggest a feasible protocol that could potentially be safely translated to clinical transplantation of lung progenitors across major MHC barriers in patients with different lung diseases.

## Supplementary Material

szab016_suppl_Supplementary_Data

## Data Availability

The data underlying this article will be shared on reasonable request to the corresponding author.
