# Supplementary material for: Lung Regeneration by Transplantation of Allogeneic Lung Progenitors Using a Safer Conditioning Regimen and Clinical-grade Reagents
Source: Stem Cells Transl Med. 2022 Feb 28;11(2):178–88. doi: 10.1093/stcltm/szab016 (PMC8929438; doi:10.1093/stcltm/szab016)
Supplement: szab016_suppl_Supplementary_Data [file szab016_suppl_supplementary_data.docx]

**Lung regeneration by transplantation of allogeneic lung progenitors using a safer conditioning regimen and clinical grade reagents.**

Irit Milman Krentsis^1,2,7^, Ran Orgad^2,7^, Yangxi Zheng^1^, Esther Bachar Lustig^1,2^, Chava Rosen^1,2,3^, Elias Shezen^1,2^, Sandeep Yadav^1^, Bar Nathansohn Levi^2^, Miri Assayag^4^, Neville Berkman^4^**,** Harry Karmouty Quintana^5^, Einav Shoshan^1^, Christa Blagdon^1^, Yair Reisner^1,2,6,8^.

**Supplementary Data**

**Table S1: Antibodies used in this study.**

| Primary antibodies | Application | Catalog number | Dilution |
| --- | --- | --- | --- |
| Rabbit anti- ERG (Abcam) | IHC | AB-92513 | 1:100 |
| Rabbit anti- Nkx2.1/ TTF1(Abcam) | IHC | AB-76013 | 1:100 |
| Rabbit anti- wide spectrum cytokeratin (Dako) | IHC | Z0622 | 1:100 |
| Rabbit anti- surfactant protein C (Santa-Cruz) | IHC | Sc-13979 | 1:100 |
| Rabbit anti- surfactant protein C (Millipore) | IHC | AB3786 | 1:200 |
| Rabbit anti- Aquaporin5 (Millipore) | IHC | 178615 | 1:150 |
| Rat anti- mouse CD31(Dianova) | IHC | DIA-310-M  Clone SZ31 | 1:50 |
| Goat anti- mouse CD31(R&D) | IHC | AF3628 | 1:200 |
| Rabbit anti- mouse HOPX (Proteintech) | IHC | 11419-1-AP | 1:200 |
| Guinea pig anti- mouse LAMP3 (SYSY) | IHC | 391005 | 1:200 |
| Rabbit anti- TdTomato (Takara) | IHC | 632496 | 1:300 |
| Goat anti- TdTomato (MyBioScience) | IHC | MBS448092 | 1:300 |
| Chicken anti- GFP (Abcam) | IHC | ab13970 | 1:500 |
| Rat anti- mouse Endomucin (Santa Cruz) | IHC | sc65495 | 1:100 |
| Rat anti- mouse Endomucin (Abcam) | IHC | ab106100 | 1:150 |
| Rat anti- mouse E-Cadherin (Invitrogen) | IHC | 131900 | 1:150 |
| Rabbit anti- CFTR (Proteintech) | IHC | 20738-1-AP | 1:150 |
| Rat anti- mouse LYVE1 (Invitrogen) | IHC | 14044382 | 1:200 |
| Hoecht 33342 (Invitrogen) | IHC | H3570 | 1:50,000 |
| Anti- mouse CD45 APC-Cy7 (Biolegend) | FACS | 103116 | 10^6^cells /1µl |
| Anti- mouse CD45 PE (Biolegend) | FACS | 103106 | 10^6^cells /1µl |
| Anti- mouse H-2K^k^ PE (Biolegend) | FACS & IHC | 114907 | 10^6^cells /1µl |
| Anti- mouse H-2K^b^ Alexa Fluor 488 (Biolegend) | FACS & IHC | 116510 | 10^6^cells /1µl |
| Anti- mouse H-2K^b^ APC (Biolegend) | FACS & IHC | 116518 | 10^6^cells /1µl |
| Anti- mouse H-2K^d^ Alexa Fluor 647 (Biolegend) | FACS & IHC | 116612 | 10^6^cells /1µl |
| Anti- mouse H-2L^d^/ H2D^b^ PE (Biolegend) | FACS & IHC | 114507 | 10^6^cells /1µl |
| Anti- mouse H-2D^d^/ H2K^d^ Alexa Fluor 647 (Biolegend) | FACS & IHC | 114712 | 10^6^cells /1µl |
| Anti- mouse CD8 Pacific Blue (Biolegend) | FACS | 100725 | 10^6^cells /1µl |
| Anti- mouse CD4 PE (Biolegend) | FACS | 100408 | 10^6^cells /1µl |
| Anti- mouse CD3 FITC (Biolegend) | FACS | 100306 | 10^6^cells /1µl |
| Ghost UV 450 (Tonbo-BioScience) | FACS | 130868T100 | 10^6^cells /2µl |
| 7-AAD (BD Pharmingen) | FACS | 51-68981E | 10^6^cells /1µl |
| **Secondary antibodies*** |  |  |  |
| Anti- chicken Alexa Fluor 488 | IHC | 703-545-155 | 1:200 |
| Anti- rabbit Rhodamine Red | IHC | 711-295-152 | 1:200 |
| Anti- rabbit Alexa Fluor 647 | IHC | 711-605-152 | 1:200 |
| Anti- rat Alexa Fluor 594 | IHC | 712-585-150 | 1:200 |
| Anti- goat Alexa Fluor 488 | IHC | 705-545-003 | 1:200 |
| Anti- goat Alexa Fluor 594 | IHC | 705-585-003 | 1:200 |
| Anti- goat AMCA | IHC | 705-155-003 | 1:200 |

*****Unless otherwise indicated, all the secondary antibodies were produced in donkey and were purchased from Jackson ImmunoResearch or Abcam.

All information regarding the antibodies, their specificity, cross-reactivity, application, and isotype controls is available on the manufacturers’ websites.


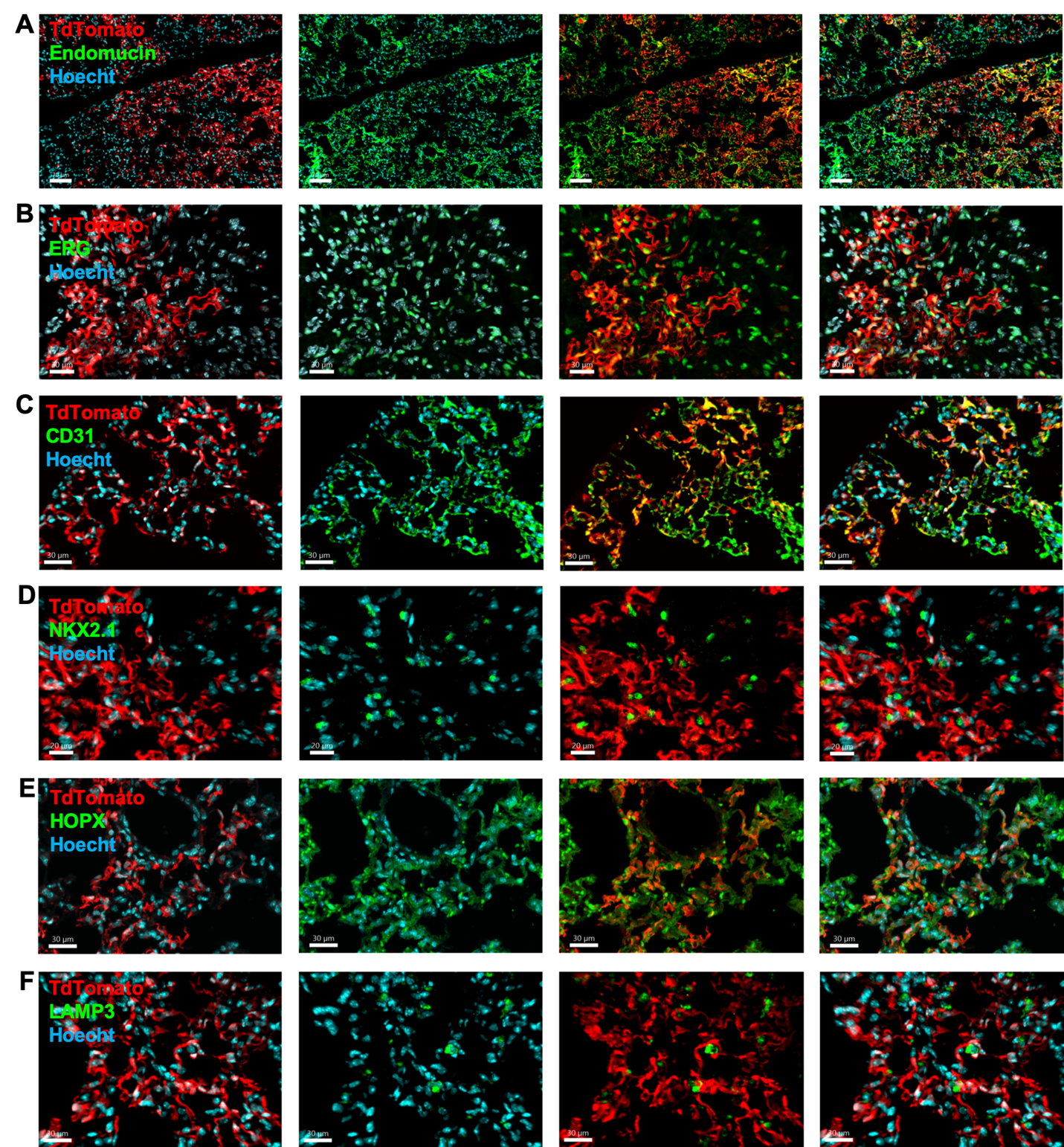


***Fig. S1: Co-localization of donor-derived lung cells****. Transplanted host lung tissue was stained for different markers to determine the distribution of the patches to different lung cell lineages- Endothelial cells were stained by Endomucin (****A****, Scale bar size=30um), ERG (****B****, Scale bar size=30um), and CD31 (****C****, scale bar size=30um). Epithelial cells were stained by the NKX2.1 marker (****D****, scale bar size=20um), HOPX (****E****, scale bar size=30um), and LAMP3 for epithelial type II cells (ATII), (****F****, scale bar size=30um); (N=2, 5-8 mice per group).*

*
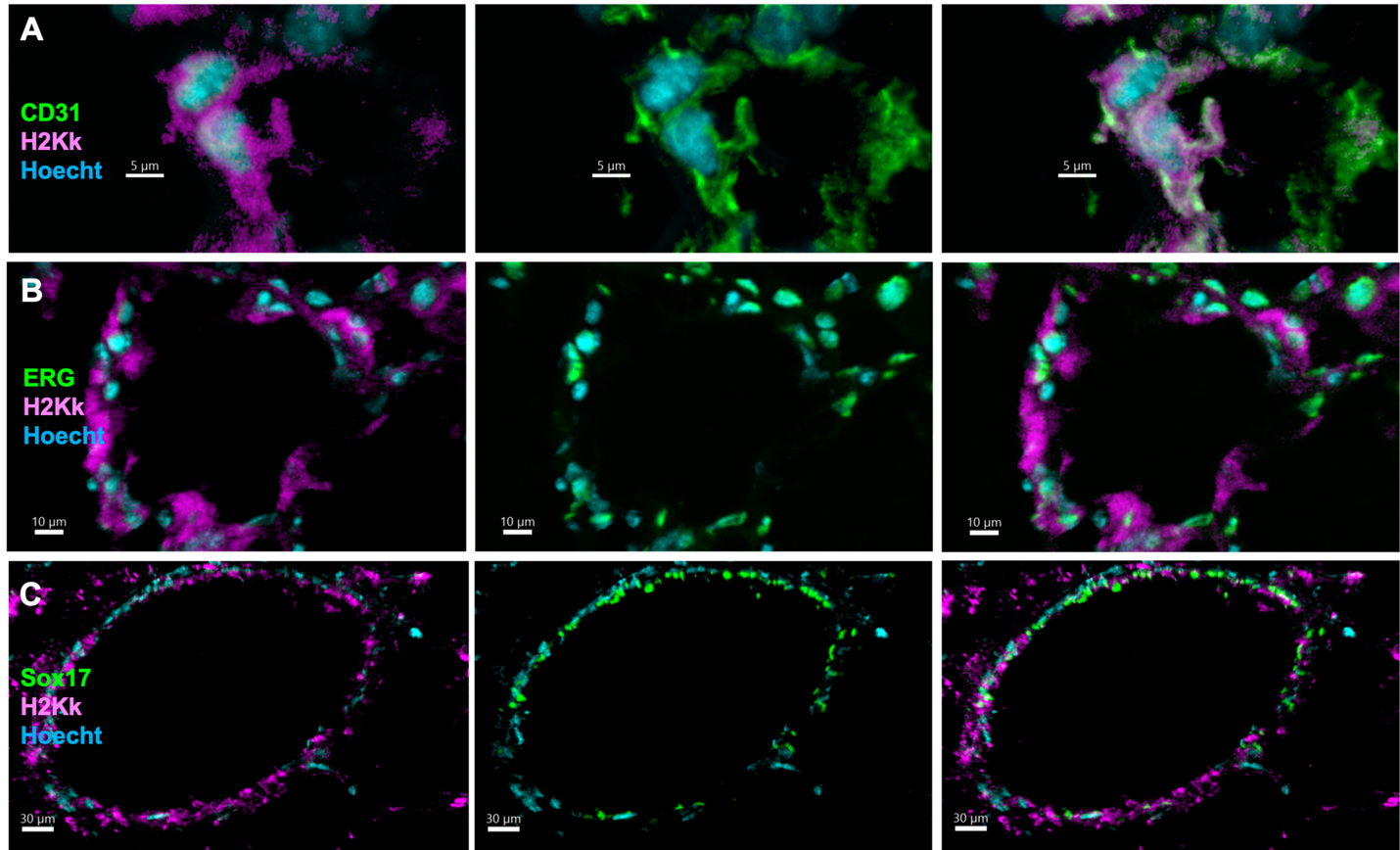
*

***Fig. S2:*** ***Representative lung immunostaining 8 weeks following transplantation of allogenic lung cells, depicting integration of donor-derived cells into endothelial compartments.*** *Donor derived lung “patches” stained for H-2K^k^ (magenta) comprise cells positive for the endothelial membranal cell markers CD31 (****A****) (Scale bar=5um), nuclear ERG (****B****) (Scale bar=10um), and Sox17 (****C****) (Scale bar=30um) (Green).*


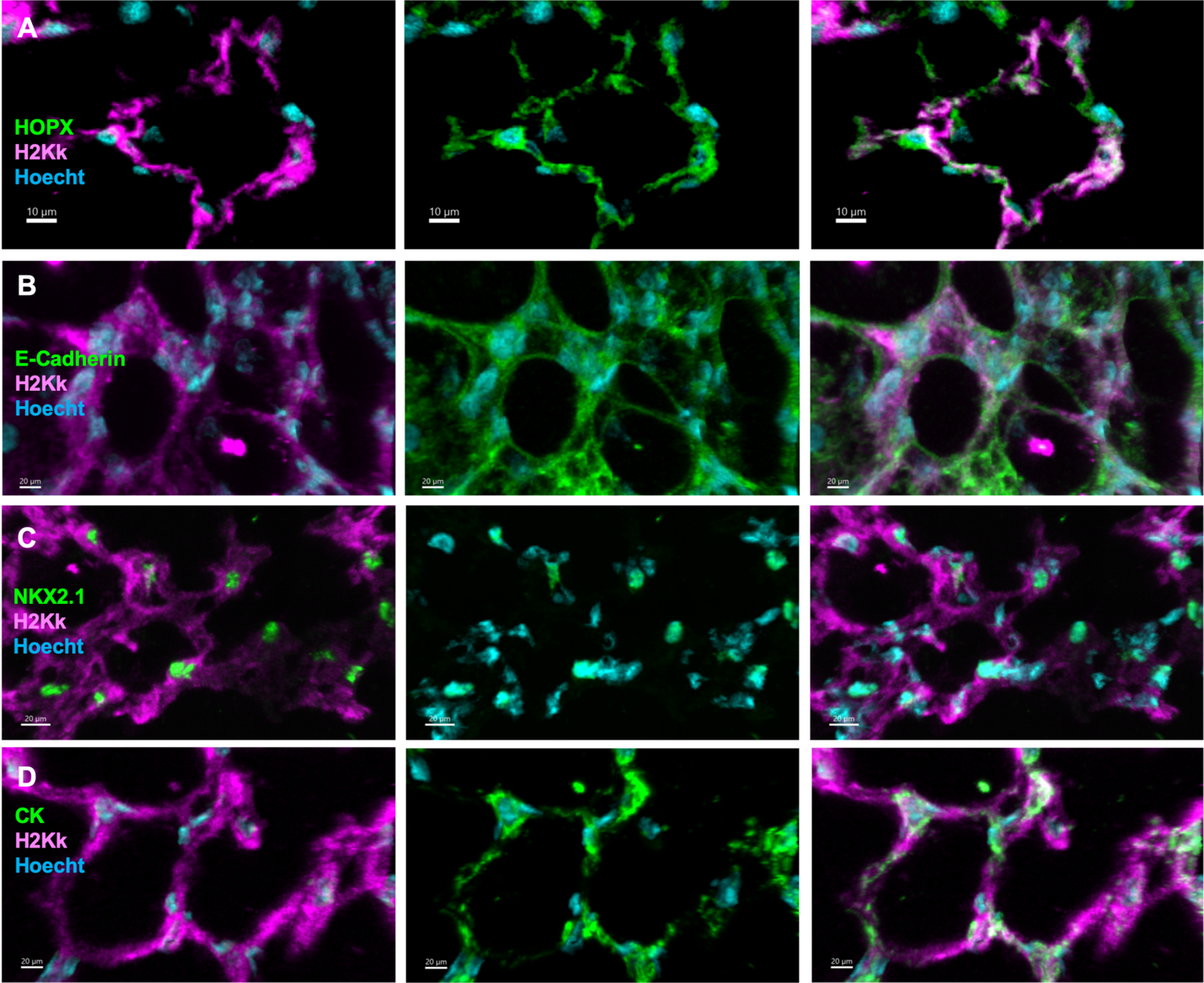


***Fig. S3:*** ***Representative lung immunostaining 8 weeks following transplantation of allogenic lung cells, depicting integration of donor-derived cells into the epithelial compartments.*** *Donor derived lung “patches” stained for H-2K^k^ (magenta) comprise cells positive for the epithelial cell markers HOPX (****A****) (Scale bar=10um), E-Cadherin (****B****), NKX2.1 (****C****), and Wide Spectrum Cytokeratin (****D****) (Scale bar=20um) (Green).*


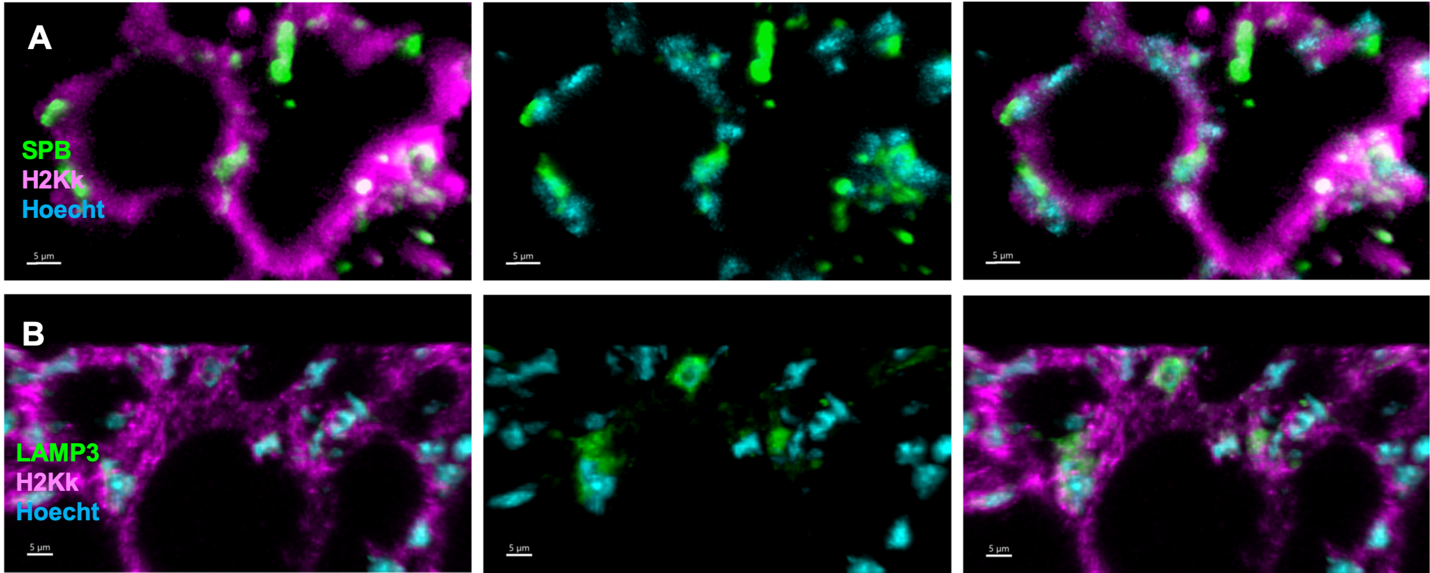


***Fig. S4:*** ***Representative lung immunostaining 8 weeks following transplantation of allogenic lung cells, depicting integration of donor-derived cells into the epithelial type II cell compartment.*** *Donor-derived lung “patches” stained for H-2K^k^ (magenta) comprise cells positive for the epithelial type II cell marker - Surfactant B (****A****), and LAMP-3 (****B****) (Scale bar=5um) (Green).*
